# Regulation of HHLA2 expression in kidney cancer and myeloid cells

**DOI:** 10.1186/s12885-023-11496-9

**Published:** 2023-10-27

**Authors:** Tomonari Shigemura, Nahuel Perrot, Zimo Huang, Rupal S. Bhatt, Aseman Bagheri Sheshdeh, Nourhan El Ahmar, Fatme Ghandour, Sabina Signoretti, David F. McDermott, Gordon J. Freeman, Kathleen M. Mahoney

**Affiliations:** 1 Department of Medical Oncology, Dana-Farber Cancer Institute, Harvard Medical School, 450 Brookline Ave., Boston, MA 02215 USA; 2 Department of Medicine, Division of Medical Oncology, Beth Israel Deaconess Medical Center, Harvard Medical School, 330 Brookline Ave, Boston, MA 02215 USA; 3 Department of Pathology, Brigham and Women’s Hospital, Harvard Medical School, 75 Francis St, Boston, MA 02115 USA

**Keywords:** HHLA2, KIR3DL3, Kidney cancer, ccRCC, Monocytes, Tumor microenvironment

## Abstract

**Background:**

The immune checkpoint HERV-H LTR-associating 2 (HHLA2) is expressed in kidney cancer and various other tumor types. Therapeutics targeting HHLA2 or its inhibitory receptor KIR3DL3 are being developed for solid tumors, including renal cell carcinoma (RCC). However, the regulation of HHLA2 expression remains poorly understood. A better understanding of HHLA2 regulation in tumor cells and the tumor microenvironment is crucial for the successful translation of these therapeutic agents into clinical applications.

**Methods:**

Flow cytometry and quantitative real-time PCR were used to analyze HHLA2 expression in primary kidney tumors ex vivo and during in vitro culture. HHLA2 expression in A498 and 786-O ccRCC cell lines was examined in vitro and in subcutaneous tumor xenografts in NSG mice. Monocytes and dendritic cells were analyzed for HHLA2 expression. We tested a range of cytokines and culture conditions, including hypoxia, to induce HHLA2 expression.

**Results:**

Analysis of HHLA2 expression revealed that HHLA2 is expressed on tumor cells in primary kidney tumors ex vivo; however, its expression gradually diminishes during a 4-week in vitro culture period. A498 and 786-O ccRCC tumor cell lines do not express HHLA2 in vitro, but HHLA2 expression was observed when grown as subcutaneous xenografts in NSG immunodeficient mice. Induction experiments using various cytokines and culture conditions failed to induce HHLA2 expression in A498 and 786-O tumor cell lines in vitro. Analysis of HHLA2 expression in monocytes and dendritic cells demonstrated that only IL-10 and BMP4, along with IL-1β and IL-6 to a lesser extent, modestly enhanced HHLA2 protein and mRNA expression.

**Conclusions:**

HHLA2 expression is induced on kidney cancer cells in vivo by a tumor microenvironmental signal that is not present in vitro. HHLA2 expression is differentially regulated in kidney cancer epithelial cells and monocytes. Cytokines, particularly IL10, that induce HHLA2 expression in monocytes fail to upregulate HHLA2 expression in tumor cell lines in vitro. These findings underscore the importance of the interplay between tumor cell and tumor microenvironmental signals in the regulation of HHLA2. Further investigation is warranted to elucidate the mechanisms involved in HHLA2 regulation and its implications for therapeutic development.

## Introduction

Immune checkpoint blockade-based therapies have revolutionized the field of cancer treatment [1–3]. Although modulation of T cell immunosuppressive and immunostimulatory functions is effective in a variety of cancer types, including melanoma, lung, bladder, and kidney cancers, a significant proportion of patients do not respond to this therapy and even those who do respond often develop resistance [4–6]. The development of therapeutic strategies targeting new immune pathways is of great importance.

Human endogenous retrovirus-H long terminal repeat-associating protein 2 (HHLA2; also known as B7-H5 and B7-H7) is a recently described ligand of the B7 family [7, 8]. Even though HHLA2 mRNA is broadly expressed in healthy human tissues [9], HHLA2 protein expression is limited, being expressed in epithelial cells of the intestine, kidney, breast, gallbladder, and placental trophoblast cells [10]. In immune cells, HHLA2 protein is reported to be expressed mainly on antigen-presenting cells; monocytes, B lymphocytes, and dendritic cells [7, 8]. On the other hand, HHLA2 protein is overexpressed in many types of human cancers, including kidney, breast, lung, thyroid, melanoma, pancreatic, ovarian, liver, bladder, colon, prostate, and esophageal cancer [10–12]. HHLA2 expression in tumor cells is generally associated with more severe disease and poor prognosis, although several studies have reported that high HHLA2 expression is associated with a favorable prognosis [13]. This discrepancy may be due to the dual role of HHLA2 as both an inhibitory and stimulatory immune checkpoint, which is not fully understood and likely to be context-dependent. Given that HHLA2 is overexpressed in various types of cancer cells and plays a heterogeneous prognostic role, it is critical to elucidate the molecular mechanisms regulating HHLA2 expression in order to best devise new strategies for targeting this pathway and selecting cancer patients who would optimally benefit.

Possible factors that regulate HHLA2 expression include transcriptional regulation, induction by inflammatory stimuli, gene copy number amplification, and epigenetic modifications [13, 14]. In monocytes and B cells, lipopolysaccharide (LPS)/IFN-γ stimulation has been reported to upregulate HHLA2 expression [8], but the regulation of HHLA2 by other factors including other cytokines remains to be investigated. In tumor cells, Wang et al. recently reported that IFN-γ upregulates HHLA2 expression in hepatocellular carcinoma [15], while Bhatt et al. reported that HHLA2 regulation and PD-L1 regulation may be distinct as HHLA2 is not induced by IFN-γ in renal cell carcinoma (RCC) cell lines [12]. Furthermore, primary human RCCs have been found to highly express HHLA2 based on immunohistochemical and RNA-Seq data from The Cancer Genome Atlas (TCGA) database [9, 10, 12, 16], while RCC cell lines rarely express HHLA2 in vitro*,* indicating a context dependent role of the tumor microenvironment in regulating HHLA2 expression. To investigate the regulation of HHLA expression, we tested a variety of factors for HHLA2 induction in RCC cell lines both in vitro and ex vivo*.* In addition, we searched for inflammatory stimuli, cytokines, and conditions that could induce HHLA2 expression on monocytes and dendritic cells.

## Materials and methods

### Cell lines and cell culture

Human renal adenocarcinoma cell lines A498 and 786-O, human lung adenocarcinoma cell line A549 and human breast cancer cell line MCF7 were purchased from the ATCC. A498, 786-O, and A549 were grown in MEM α (Life Technologies; cat. #12,571,063), RPMI-1640 (Life Technologies; cat. #11,875,093), and Ham's F-12 K (Life Technologies; cat. #21,127,022) media, respectively, supplemented with 10% fetal bovine serum (FBS; Sigma-Aldrich; cat. #F2442), 1% Gluta-Max (Life Technologies; cat. #35,050–061), 1% penicillin/streptomycin (Gibro; cat. #15,140,122), and 15 μg/mL gentamicin (Gibco; cat. #15,710,064). MCF7 was cultured in Eagle's Minimum Essential Medium (Sigma-Aldrich; cat. #M4655) with 10% FBS, 1% Gluta-Max, 1% penicillin/streptomycin, 15 μg/mL gentamicin, and 0.01 mg/mL human recombinant insulin (Sigma-Aldrich; cat. #I9278). All cell lines were maintained at 37°C with 5% CO_2_ and not kept in culture for longer than 4 months, before which an aliquot of the original stock was thawed. Further, depending on the experiment, these cell lines were cultured in glucose-depleted medium; RPMI 1640 without glucose (Sigma-Aldrich; cat. #11,879,020) supplemented with 10 mM galactose (Sigma-Aldrich; cat. #G0750), 10% FBS and 1% Gluta-Max, or lactic acidosis condition; 20 mM lactate (Sigma-Aldrich; cat. #71,718) with acidity regulated by hydrochloric acid (pH 6.5–6.7), or demethylated with 10 μM 5-Aza-2’-deoxycytidine (Sigma-Aldrich; cat. #189,826) for 4 days, or cultured under hypoxia-inducing conditions with 100 μM Cobalt(II) chloride hexahydrate (CoCl_2_) (Sigma-Aldrich; cat. #C8661) for 1 day, or with the indicated cytokines.

### Cytokines and growth factors

Cytokines and growth factors were purchased from the indicated companies and used at the final concentrations indicated in Table 1. For the IFN-α, IFN-β, IFN-γ, IL-1β, IL-6, IL-10, and IL-15 combination experiments, the cytokine concentrations shown in Table 1 were used.

**Table 1:** Cytokines and growth factors tested for HHLA2 induction

**Set-1**	**Concentration**			**Company**	**cat. #**
IFN-alpha A (alpha 2a)	25,000	IU/ml	human	R&D Systems	11,100–1
IFN-β	50	ng/ml	human	peprotech	300-02BC
IFN-γ	50	ng/ml	human	peprotech	300–02
IL-1β	100	ng/ml	human	peprotech	200-01B
IL-2	100	ng/ml	human	peprotech	200–02
IL-4	100	ng/ml	human	peprotech	200–04
IL-6	100	ng/ml	human	peprotech	200–06
IL-7	100	ng/ml	human	peprotech	200–07
IL-8	250	ng/ml	human	peprotech	200-08 M
IL-9	100	ng/ml	human	peprotech	200–09
IL-10	100	ng/ml	human	peprotech	200–10
IL-13	100	ng/ml	human	peprotech	200–13
IL-15	100	ng/ml	human	peprotech	200–15
IL-21	100	ng/ml	human	peprotech	200–21
IL-22	100	ng/ml	human	peprotech	200–22
IL-27	100	ng/ml	human	peprotech	200–38
IL-33	100	ng/ml	human	peprotech	200–33
IL-34	100	ng/ml	human	peprotech	200–34
BMP2	1,000	ng/ml	human	peprotech	120-02C
BMP4	1,000	ng/ml	human	peprotech	120-05ET
BMP6	1,000	ng/ml	human	peprotech	120–06
BMP7	1,000	ng/ml	human	peprotech	120-03P
GDF-2 (BMP9)	100	ng/ml	human	peprotech	120–07
BMP10	1,000	ng/ml	human	peprotech	120–40
BMP4/7 Heterodimer	1,000	ng/ml	human	peprotech	3727-BP-010/CF
TNF-α	50	ng/ml	human	peprotech	300-01A
**Set-2**	**Concentration**			**Company**	**cat. #**
IL-18	3,000	ng/ml	human	R&D Systems	9124-IL
TGF-β1β	50	ng/ml	human	peprotech	100–21
GDF15	2,000	ng/ml	human	peprotech	120–07
LIF	250	ng/ml	human	peprotech	300–05
Oncostatin M (227 a.a.)	1,000	ng/ml	human	peprotech	300–10
HGF	1,000	ng/ml	human	peprotech	100–39
IGF-I	100	ng/ml	human	peprotech	100–11
VEGF165	100	ng/ml	human	peprotech	100–20
PDGF-AB	200	ng/ml	human	peprotech	100-00AB
EGF	500	ng/ml	human	peprotech	AF-100–15
Dexamethasone	10	μM		R&D Systems	1126/100
1,25-Dihydroxyvitamin D3	100	ng/ml		Supelco	H-107-1ML
Pam3CSK4 (TLR1/2)	5	μg/ml		R&D Systems	4633/1
Poly(I:C) (TRL3)	12.5	μg/ml		R&D Systems	4287/10
Lipopolysaccharide (TLR4)	5	μg/ml		Invitrogen	00–4976-93
Imiquimod (TLR7)	5	μg/ml		R&D Systems	3700/50
**Concentration in cytokine combination experiments**					
IFN-α	12,500	IU/ml	IL-18	1,000	ng/ml
IFN-β	12.5	ng/ml	IL-21	50	ng/ml
IFN-γ	20	ng/ml	IL-22	50	ng/ml
IL-1β	20	ng/ml	BMP2	500	ng/ml
IL-6	50	ng/ml	BMP4	500	ng/ml
IL-10	50	ng/ml	GDF15	400	ng/ml
IL-15	50	ng/ml	HGF	500	ng/ml

Source and final concentrations of cytokines and growth factors used to stimulate monocytes, dendritic cells, and ccRCC cell lines

### Tumor collection, processing, and establishment of RCC cell lines

RCC samples were obtained from Beth Israel Deaconess Medical Center (BIDMC) from patients providing written consent under Dana Farber/Harvard Cancer Center (DF/HCC) institutional review board (IRB)–approved Renal cancer tissue collection protocol 01–130. Tumor tissue from surgical resections was minced using a scalpel, then incubated in digestion media, consisting of 0.11 Units/mL collagenase D (Roche; cat. #11,088,858,001), 0.56 Units/mL dispase (STEMCELL; cat. #7913), 50 Units/mL DNase I (New England; cat. #M0303L), 5 mM calcium chloride (VWR; cat. #E506-100ML), and HBSS (Gibco; cat. #14,175,095) at 37 °C for 10 min with occasional stirring. Remaining undissociated tissue and cell clumps were filtered out using 100 μM cell strainer (Fisher Scientific cat. #14,175,095). Contaminating red blood cells were lysed with 1X RBC Lysis Buffer (eBioscience; cat. #00–4333-57). Primary RCC cell lines were generated by isolating carbonic anhydrase 9 (CA9)-positive cells from patient tumor specimens as previously described [17–19] using recombinant anti-CA9-PE antibody (Miltenyi; cat. #130–123-299) and anti-PE Microbeads Ultrapure (Miltenyi; cat. #130–105-639). Cells were positively selected by separation over two LS Columns (Miltenyi; cat. #120–042-401). CA9-positive cells were cultured in tissue culture plates containing OptiMEM GlutaMax media (Gibco; cat. #51,985,034) supplemented with 5% FBS, 1 mM sodium pyruvate (Gibco; cat. #11,360,070), 1% penicillin/streptomycin, 15 μg/mL gentamicin, 5 µg/mL insulin and 5 ng/ml epidermal growth factor (Peprotech; cat. #AF-100–15). Day 0 in culture was defined as when cells were first plated in culture, which given processing time was within 24 h of the tumor being removed from the patient in the setting of their nephrectomy. Cell cultures were dissociated and passaged using versene (Gibco; cat. #15,040,066).

### Growth of ccRCC cell lines in NSG mice

Five million A498 cells were injected subcutaneously into the left flank of NSG mice. Tumors were harvested once they reached a size of at least 13 mm in length. Tumor tissues were digested with tumor dissociation kits (Miltenyi, cat.#130,095,929) for 30 min at 37°C. Remaining undissociated tissue and cell clumps were filtered out using a 70 μm cell strainer (Falcon, cat.#352,350). A498 tumors were analysed the day of harvest from the mouse or after culture in vitro in MEMα media as described above for up to 14 days. Cells were stained with Zombie Violet Live/Dead dye (Biolegend, cat.#423,114), PE-conjugated anti-CD70 (Biolegend, cat. #355,104), and Alexa 647-conjugated anti-HHLA2 antibody 6F10. All procedures involving animals were reviewed and approved by the Institutional Animal Care and Use Committee of the Beth Israel Deaconess Medical Center (IACUC protocol number: 085–2020).

### Immunohistochemistry of ccRCC xenografts in NSG mice

Immunohistochemical staining was performed on formalin-fixed, paraffin-embedded 786-O and A498 tumor xenograft tissue sections using a rabbit monoclonal HHLA2 antibody (Cell Signaling Technology, clone: E1U6X, 1:100 dilution), a rabbit monoclonal anti-mouse CD45 antibody (Cell Signaling Technology, clone D3F8Q, 1:100 dilution), and a rabbit monoclonal anti-mouse F4/80 antibody (Cell Signaling Technology, clone D2S9R, 1:500 dilution). The assay was developed using formalin-fixed, paraffin-embedded cell line controls demonstrated to be either positive or negative for HHLA2 expression by flow cytometry (Supplementary Fig. 1). Briefly, 5 µm thick tissue sections were mounted on charged slides and placed for 30 min in the oven at 60°C degrees. Deparaffinization and immunostaining was performed using the Bond III autostainer (Leica Biosystems) and the Bond Polymer Refine Detection Kit (DS9800, Leica Biosystems) according to the manufacturer’s guidelines. Antigen retrieval was performed with Bond Epitope Retrieval Solution 1 (Citrate, pH = 6) for 30 min for all three antibodies. All slides were counterstained with hematoxylin, dehydrated in graded ethanol and xylene, and coverslipped. The percentage of tumor cells with membranous HHLA2 expression was independently assessed in each tumor by two pathologists (NSA and FG). Interscorer discrepancies were resolved by consensus review.

### Human monocyte isolation and culture

RosetteSep Human monocyte Cell Enrichment Cocktail (Stemcell; cat. #15,028) was used according to the manufacturer’s protocol to isolate monocytes by negative selection from the blood of anonymous healthy donors. Donors signed a clinical consent form for the donation procedure, which includes language that the donor center can direct use of all materials and by-products for clinical or research use. All procedure involving peripheral blood mononuclear cells (PBMCs) from anonymous donors were reviewed and approved in DF/HCC IRB-exempted protocol 93–011. Monocytes were seeded in 96-well flat bottom plates (Costar; Cat. #3596) in X-VIVO 15 medium (Lonza; Cat. #04418Q) at 0.6 × 10^6^/well and different cytokines were added.

### Preparation of monocyte-derived DCs

DCs were prepared from blood monocytes according to previously established protocols [20] with some modifications. Monocytes were cultured in 6-well plates at 3 × 10^6^ cells/well (Falcon; cat. #353,046) in X-VIVO 15 and supplemented with 50 ng/ml IL-4 and 80 ng/mL GM-CSF (Peprotech; cat. #300–03) and incubated for 5 days to generate immature DCs (iDCs). These iDCs were seeded at 0.5 × 10^6^ cells/well in 24-well plates (Falcon; cat. #353,047) in IL-4 (50 ng/mL) and GM-CSF (80 ng/mL) plus IL-1β, IL-6, IL-10 or IL-10 + IFN-γ, or further stimulated for 2 days with IL-1ꞵ (20 ng/mL), TNF-α (10 ng/mL), IL-6 (50 ng/mL), and prostaglandin E2 (R&D Systems; cat. #2296/10) (1 µg/mL) to generate mature DCs (mDCs). Where indicated, IL-10 (50 ng/mL) or IL-10 + IFN-γ (20 ng/mL) was added to the maturation culture medium for 2 days (Fig. 4A, B).

### FACS analysis for expression of HHLA2 on monocytes, DCs, and RCC cells

At the indicated times, monocytes and DCs were stained with the following antibodies after blocking Fc receptors (Biolegend; cat. #422,302): Alexa 647-conjugated anti-HHLA2 antibody, clone 499.6F10, generated as described [12] or Alexa 647-conjugated mouse IgG1, k isotype control (BioLegend; cat. #400,130) at 5 µg/mL; BV421-conjugated anti-human CD14 (BioLegend; cat. #367,144) for monocytes; BV421-conjugated anti-human CD11c (BioLegend; cat. #371,512) for DCs. RCC cell lines and primary RCC cells were also stained with Alexa 647-conjugated HHLA2 antibody at the same concentration. In the co-culture experiment, FITC-conjugated anti-human CD70 (BioLegend; cat. #355,106) was used as a tumor marker for RCC. Cells were analyzed for HHLA2 expression in CD14^+^ gated cells for monocytes, CD11c^+^ gated cells for DCs, and CD70^+^ gated cells for RCC cells in co-cultures using flow cytometry.

### Flow cytometry

Cells were analyzed on a BD LSRFortessa X-20 Cell Analyzer, BD LSRFortessa Cell Analyzer, or Beckman Coulter CytoFLEX LX Cytometer. For each experiment, 10,000 to 20,000 cells were analyzed. Data were analyzed with FlowJo software.

### RNA extraction and quantitative real-time PCR

Total RNA was extracted from cell pellets using Purelink RNA mini Kit (Thermo Fisher) or RNeasy mini kit (QIAGEN) as per the manufacturer’s protocol. cDNA synthesis was performed with the High Capacity RNA-to-cDNA Kit (Applied Biosystems, Thermo Fisher) according to the manufacturer’s recommendations. RT-PCR was performed in Applied Biosystems™ QuantStudio™ 6 Flex Real-Time machine using Power SYBR Green PCR Master Mix according to the manufacturer’s recommendations. Primers were designed in the lab and ordered as DNA oligos from Thermo Fisher. The primer sequences are shown below: 18S, forward: 5’-GTA ACC CGT TG AAC CCC ATT-3’, reverse, 5’- CCA TCC AAT CGG TAG TAG CG-3’; HHLA2, forward, 5’-TAC AAA GGC AGT GAC CAT TTG G-3’, reverse, 5’- AGG TGT AAA TTC CTT CGT CCA GA-3’.

18S rRNA was used as internal control for each sample. Relative mRNA levels were determined by the 2-∆∆CT formula, and experiments were repeated three times.

### Statistical analysis

GraphPad Prism statistics version 9.0 software (GraphPad Software Inc., San Diego, CA, USA) was used for analysis and data graphing, unless otherwise indicated. An unpaired Student’s t-test was used for comparing two groups or one-way ANOVA for comparisons with more than two groups followed by Dunnett's multiple comparisons test for comparison with the control group as indicated in the legends [nonsignificant (ns), *p < 0.05, **p < 0.01, ***p < 0.001, and ****p < 0.0001]. All data are expressed as mean ± SEM.

Data Sharing Statement: For original data, please contact: Gordon_freeman@dfci.harvard.edu.

## Results

### RCC tumor cells progressively lose HHLA2 expression during in vitro culture

**Fig. 1:**
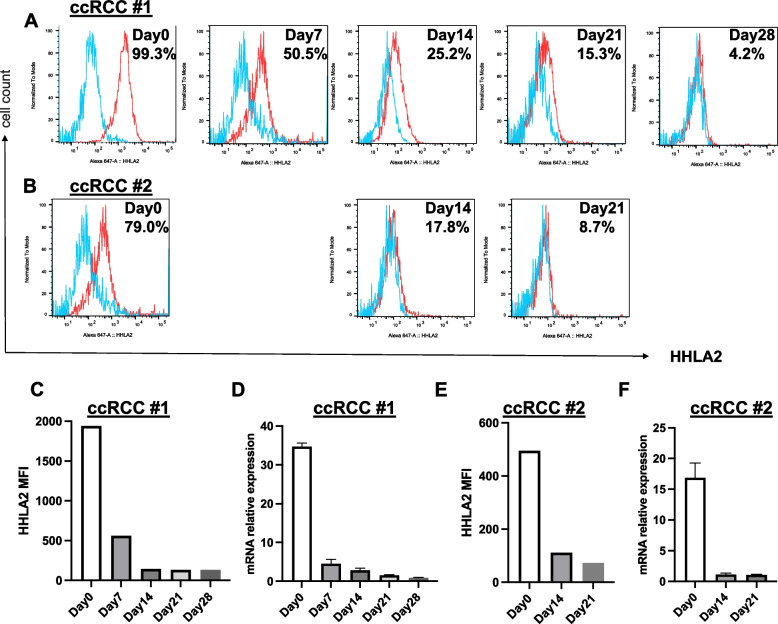
Progressive loss of HHLA 2 expression after culture of RCC cells in vitro. Surgically removed RCC tissue was dissociated into single-cells and assessed for HHLA2 expression using flow cytometry ex vivo and after the indicated times of culture in vitro (**A**,** B**,** C**,** E**) (*n* = 10 ccRCC patients) and by qPCR for mRNA (**D**,** F**) (*n* = 2 ccRCC patients). Representative histograms showing HHLA2 expression at day 0, 7, 14, 21, 28 or at day 0, 14, 21 of in vitro culture (*n* = 2 ccRCC patients). Numbers in upper right indicate % HHLA2 positive

Previous studies have reported that HHLA2 is overexpressed in many ccRCC in situ [9, 10, 12, 16, 19]. ccRCC tissues from patient nephrectomy specimens were dispersed into single-cell suspensions and analyzed for HHLA2 expression after CA9 positive selection for tumor cell purification. We confirmed that primary ccRCC tumors from nephrectomy specimens show expression of HHLA2 by flow cytometry (Fig. 1A, B and Supplemental Table 1). Ten of fifteen primary ccRCC were successfully cultured in vitro, and HHLA2 expression was observed to gradually decline and almost disappear after 2–3 weeks (Fig. 1A, B, C, E, and Supplemental Table 1). Similarly, expression of HHLA2 mRNA also declined after in vitro culture (Fig. 1D, F). The loss of HHLA2 expression on dissociated, in vitro cultured tumor cells indicates a role for a tumor microenvironmental signal that is needed to maintain HHLA2 expression.

### ccRCC tumor cell line express HHLA2 when re-introduced in vivo

**Fig. 2:**
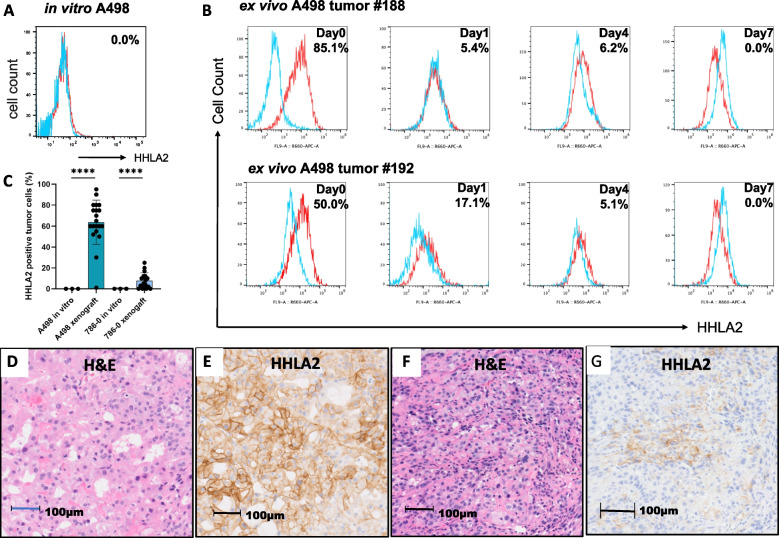
HHLA2 expression is induced on A498 ccRCC cell line in vivo. **A** Expression of HHLA2 on A498 cells in vitro*.* **B**-**G** Five million A498 or 786-O cells were injected subcutaneously into NSG mice and tumors were harvested at a size of at least 10 mm. HHLA2 expression was analyzed by (**B**) flow cytometry on day 0 and after 1, 4, and 7 days of culture in vitro (numbers in upper right indicate % HHLA2 positive) and (**C**) by immunohistochemistry on day 0. **D**-**G** representative H&E and HHLA2 images of A498 and 786-O tumors, respectively. The statistical significance of difference versus control cells is indicated as ****p < 0.0001

Despite HHLA2 expression on many ccRCC in situ, A498 and 786-O ccRCC tumor cell lines do not express HHLA2 in vitro (Fig. 2A, Supp Fig. 2A-B, 3). Immunohistochemistry of cell blocks of A498 and 786-O confirmed a complete lack of HHLA2 expression in vitro (Supp Fig. 4). We re-introduced these ccRCC cell lines in vivo in immunodeficient NSG mice. When tumors were 1.0 to 1.5 cm in size, we prepared single cell suspensions and examined tumor cells by flow cytometry. HHLA2 expression was clearly induced on A498 by these in vivo conditions (Fig. 2B). HHLA2 expression was examined by immunohistochemistry and was detected on approximately 64% and 8% of A498 and 786-O tumor cells, respectively (Fig. 2C-G). While NSG mice lack an adaptive immune system, they have residual murine myeloid cells (murine CD45 + and F4/80 +) [21, 22] which infiltrate both A498 and 786-O xenografts (Supp Fig. 5). This result shows that human ccRCC cell lines retain the capacity to respond to some in vivo signal that induces HHLA2 expression. Moreover, similar to the results with ccRCC nephrectomy specimens, HHLA2 expression on A498 was lost when tumor cells were cultured in vitro (Fig. 2B).

**Fig. 3:**
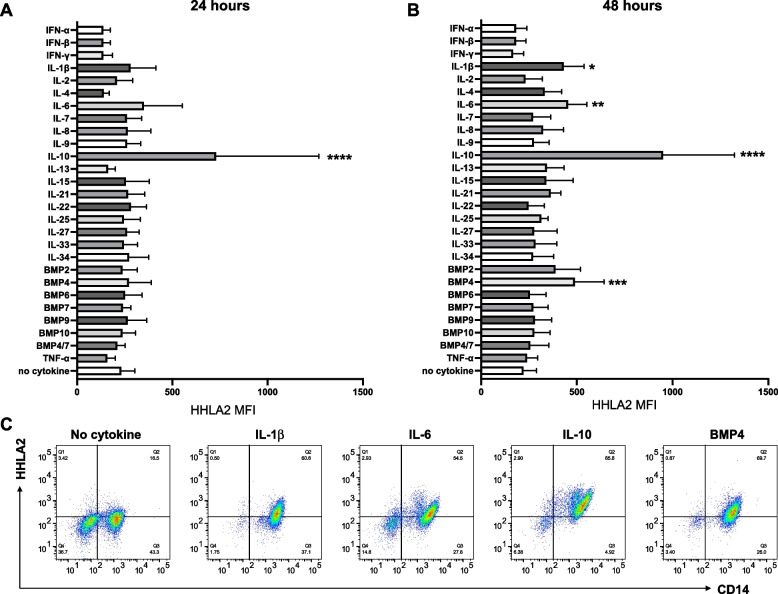
Effect of cytokines on HHLA2 expression in human monocytes. Monocytes from healthy donors were treated with the indicated cytokines and HHLA2 expression was assessed by flow cytometry at **A** 24 h and **B** 48 h. MFI = Mean fluorescence intensity. **C** Representative flow cytometry plots of cell-surface HHLA2 at 48 h. The statistical significance of difference versus no cytokine (control) is indicated as *p < 0.05, **p < 0.01, ***p < 0.001 and ****p < 0.0001. (*n* = 10 healthy donors)

**Fig. 4:**
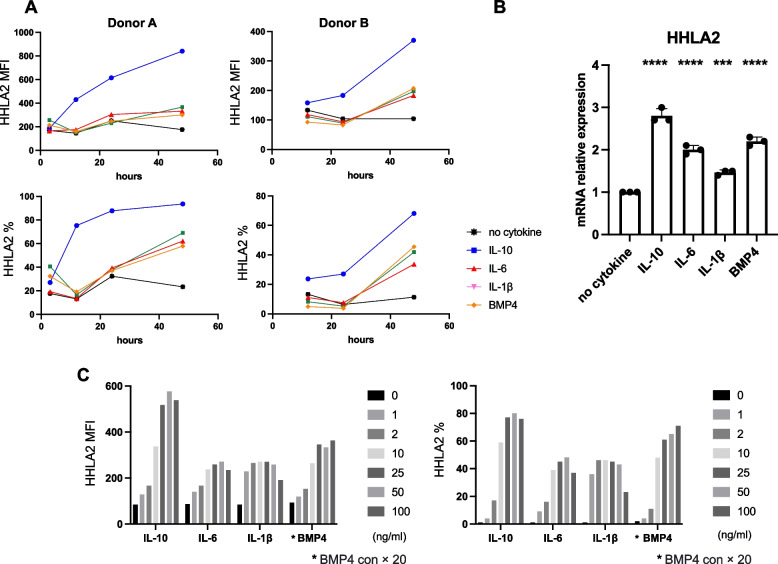
Effects of IL-10, IL-1β, IL-6 and BMP4 on HHLA2 expression in monocytes. **A** Monocytes from two donors (donor A; left and donor B; right) were treated with IL-10, IL-1β, IL-6 and BMP4, and HHLA2 expression was assessed over a time course using flow cytometry and measuring MFI and percentage of HHLA2 positivity in CD14^+^ cells (*n* = 2 healthy donors). **B** Relative HHLA2 mRNA levels were measured by qPCR at 18 h after cytokine treatment in monocytes. *N* = 3 replicates. **C** Monocytes were treated with cytokines (IL-10, IL-6, IL-1β) at 1, 2, 10, 25, 50, 100 ng/mL and BMP4 at 20, 40, 200, 500, 1000, 2000 ng/mL, and surface HHLA2 expression was measured at 48 h (*n* = 1 healthy donor)

**Fig. 5:**
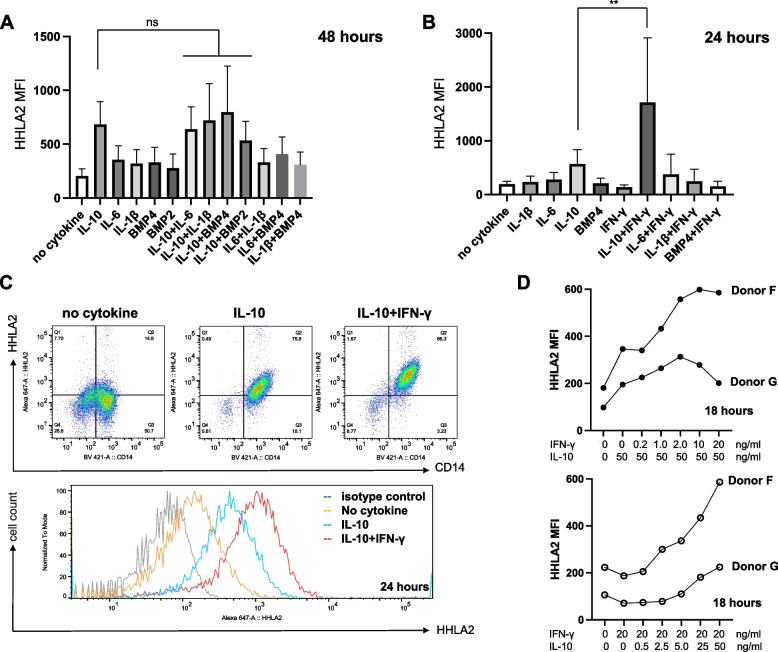
Synergistic effects of IL-10 and IFN-γ on HHLA2 induction in monocytes. **A** HHLA2 expression in monocytes was measured at 48 h using flow cytometry after treatment individually or with the indicated combinations of IL-10, IL-6, IL-1β, BMP2, and BMP4. No significant difference in IL-10 alone (control) versus combination with IL-10. (*n* = 11 healthy donors). **B** Combination effect of IL-10, IL-6, IL-1β, and BMP4 with IFN-γ was evaluated using flow cytometry at 24 h. (*n* = 6 healthy donors). **C** Representative flow cytometry plots and histograms of HHLA2 expression at 24 h. Cytokine concentrations used in the combination experiments in A, B, and C are shown in Table 1. **D** Monocytes were treated with IL-10 (50 ng/mL) plus IFN-γ at 0, 0.2, 1, 2, 10, 20 ng/mL (top) or IFN-γ (20 ng/mL) plus IL-10 at 0, 0.5, 2.5, 5, 25, 50 ng/mL (bottom). HHLA2 expression was measured as MFI at 18 h by flow cytometry (*n* = 2 healthy donors)

### Cytokine stimulation does not induce HHLA2 expression in RCC cell lines, nor do conditions of glucose deprivation, lactic acidosis, hypoxia, or demethylation

A498 and 786-O ccRCC tumor cell lines were tested with an extensive set of candidate cytokines, including a combination of IL-10 and IFN-γ; however, none induced HHLA2 expression in these cell lines (Table 2). We considered that additional factors were necessary for HHLA2 induction in RCC tumors. To mimic aspects of the tumor microenvironment, A498 and 786-O were cultured with hypoxia induction by CoCl_2_, or cultured under lactic acidosis or in glucose-free media in which glucose was replaced by galactose, but these conditions, alone or in combination with cytokines, did not induce HHLA2 (Table 2). Wang et al. pointed out that epigenetic modifications such as DNA hypomethylation may be involved in the upregulation of HHLA2 in RCC [9]. Since methylation of the promoter region of the HHLA2 gene may suppress HHLA2 expression, we attempted to induce HHLA2 expression by demethylation with 5'-azacytidine or a combination of demethylation and cytokines, but no HHLA2 expression was observed (Table 2).

**Table 2:** Culture conditions tested for HHLA2 induction

	**A498**						
	w/o cytokine	IL-10	IL-10 + IFN-γ	IFN-γ	IL-6	IL-1β	BMP4
Standard culture medium	no	no	no	no	no	no	no
Demethylation (5-Aza 5 μM)	no	no	no	no	no	no	no
Lactate (20 mM) and Acidity (pH 6.5–6.7)	no	no	no	no			
Hypoxia (CoCl2 100 μM)	no	no	no	no		no	
Glucose-deprivation, plus galactose	no	no	no	no			
	**786-O**						
	w/o cytokine	IL-10	IL-10 + IFN-γ	IFN-γ	IL-6	IL-1β	BMP4
Standard culture medium	no	no	no	no	no	no	no
Demethylation (5-Aza 5 μM)	no	no	no	no	no	no	no
Lactate (20 mM) and Acidity (pH 6.5–6.7)	no	no	no	no			
Hypoxia (CoCl2 100 μM)	no	no	no	no		no	
Glucose-deprivation, plus galactose	no	no	no	no			

A498 and 786-O ccRCC tumor cell lines were cultured in standard culture medium or demethylated with 5'-azactidine, or cultured under lactic acidosis conditions (pH 6.5–6.7), or hypoxia-inducing conditions with CoCl_2_, or in glucose-depleted medium with galactose replacement. Each of these conditions was tested plus different cytokines (IL-10, IFN-γ, IL-6, IL-1β, BMP4, or IL-10 + IFN-γ). HHLA 2 expression was assessed by flow cytometry after 48–96 h. “no” means no HHLA2 induction was seen. *n* ≥ 3 replicates

### IL-10, IL-1ꞵ, IL-6 and BMP4 induce cell surface HHLA2 expression in monocytes and up-regulate HHLA2 mRNA transcription

Human CD14^+^ monocytes in PBMCs can express HHLA2, and it has been previously reported that HHLA2 expression is further induced by combined LPS and IFN- γ stimulation [8]. Therefore, to elucidate the detailed mechanism of regulation of HHLA2 expression by inflammatory stimuli, we examined the surface expression of HHLA2 in monocytes following incubation with a variety of cytokines and growth factors. Screening of monocytes from 10 healthy donors with the 26 cytokines of set-1 (Table 1) revealed that IL-10 up-regulated HHLA2 expression most significantly. IL-6, IL-1β, and BMP4 showed no induction of HHLA2 at 24 h, but clearly induced HHLA2 at 48 h after stimulation (Fig. 3A, B, C). When expression was assessed over a time course in two donors (donors A and B), induction of HHLA2 by IL-10 was observed by 12 h and progressively increased thereafter. In contrast, IL-6, IL-1β, or BMP4 showed no obvious induction of HHLA2 at 24 h but clear induction at 48 h compared to controls (no cytokine) (Fig. 4A). Treatment with these four cytokines induced HHLA2 mRNA expression as well as protein expression (Fig. 4B). The induction of HHLA2 by each of the four cytokines was concentration dependent, reaching a plateau at 50 ng/ml for IL-10 and IL-6, 10 ng/ml for IL-1ꞵ, and 500 ng/ml for BMP4 (Fig. 4C). In contrast, IFN-γ alone did not induce HHLA2, but rather decreased its expression. Stimulation of Toll like receptor 4 by LPS also induced HHLA2 (Supp Fig. 6A, B). No other significant induction by external stimuli, including growth factors, vitamin D3, or steroid hormones, was observed. In subsequent experiments IL-10, IL-1β, IL-6, and BMP4 were used at concentrations of 50 ng/mL, 20 ng/mL, 50 ng /mL, and 500 ng/mL, respectively.

**Fig. 6:**
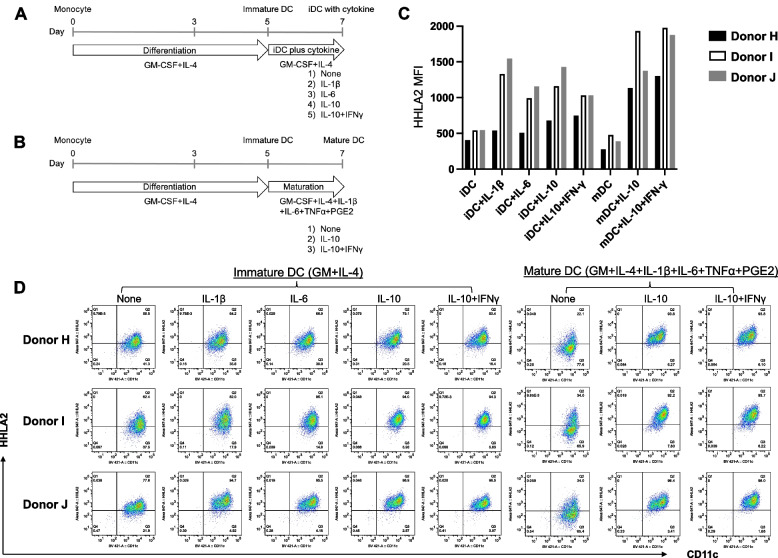
Effect of cytokines on HHLA2 expression in dendritic cells. **A**, **B** Protocol for generation and cytokine treatment of dendritic cells. Monocytes from PBMCs were cultured in GM-CSF and IL-4 for 5 days to generate immature DCs (iDC). To induce mature DCs (mDC), cells were cultured with IL-6, IL-1β, TNFα, and PGE2 as well as GM-CSF and IL-4 for an additional 2 days. **C** HHLA2 expression on iDC additionally treated with IL-1β, IL-6, IL-10, or IL-10 + IFN-γ for 2 days or on mDC treated with IL-10 or IL-10 + IFN-γ during the 2 day differentiation process into mDC. Surface HHLA2 was measured by MFI in CD11c.^+^ cells using flow cytometry. **D** Flow cytometry plots of HHLA2 expression at 48 h after cytokine addition to iDC or mDC cultures (*n* = 3 healthy donors)

### IFN-γ enhances HHLA2 induction by IL-10 in monocytes

Next, we evaluated whether these cytokines had synergistic effects on HHLA2 expression in monocytes. The combination of BMP4 or IL-1β with IL-10 slightly increased HHLA2 expression on monocytes at 48 h but this effect was not statistically significant compared with IL-10 alone. (Fig. 5A). The BMP4 and IL-10 combination showed an increase in 4 of 8 donors but one showed a lower expression than IL-10 alone. The IL-1β and IL-10 combination showed an increase in 2 of 8 donors.

Recently, Wang et al. showed that HHLA2 can be upregulated by IFN-γ in hepatocellular carcinoma [15]. Although IFN-γ alone did not induce HHLA2 in our monocyte studies, we considered the possibility that HHLA2 expression could be enhanced by IFN-γ in combination with other cytokines, since IFN-γ and IL-10 cooperatively regulated myeloid cell function in the study by Yanagawa et al. [23]. Therefore, we investigated the combination of IFN-γ and additional cytokines on HHLA2 induction. IL-10 had a synergistic effect with IFN-γ, with a threefold higher MFI than IL-10 alone. IFN-γ in combination with other candidate cytokines did not increase HHLA2 expression (Fig. 5B, C; Supp Fig. 7A). The increase in HHLA2 expression in response to the combination of IL-10 with IFN-γ was also observed with the Type 1 interferons, IFN α- and IFN-β (Supp Fig. 7B). IL-10 alone did not induce much HHLA2 at 24 h in donor E, but in combination with IFNs induced HHLA2 even in monocytes of such an unresponsive donor (Supp Fig. 7C). The HHLA2-inducing effect of the IL-10/IFN-γ combination did not increase above 2 ng/ml IFN-γ but higher levels of IL-10 were able to increase HHLA2 expression in the presence of a constant amount of IFN-γ (Fig. 5D). These results indicate that lower levels of IFN-γ are sufficient to enhance the effect of HHLA2 induction by IL-10, suggesting that IFN-γ may be an important cytokine in HHLA2 induction in monocytes.

### IL-10 maintains HHLA2 expression in mDC and IFN-γ enhances its effect

In the report by Zhao et al. [8], no HHLA2 protein expression was found in monocyte-derived dendritic cells (DCs). However, in our study, when immature DCs (iDCs) were induced from peripheral blood monocytes with GM-CSF and IL-4 (Fig. 6A), HHLA2 expression was observed in all three healthy donors (Fig. 6C, D). Furthermore, HHLA2 expression was enhanced in these iDCs by IL-1β, IL-6, IL-10, or IL-10 plus IFN-γ added to GM-CSF + IL-4. Mature DCs (mDCs) were induced by two days further culture with GM-CSF + IL-4 plus TNF-α, IL-1β, IL-6, and prostaglandin E2 (PGE2) according to established protocols [20] (Fig. 6B) but showed attenuated HHLA2 expression compared to iDC (Fig. 6C, D). Addition of IL-10 during mDC induction enhanced HHLA2 expression and the combination of IL-10 and IFN-γ tended to further enhance it (Fig. 6C, D).

## Discussion

HHLA2 and its inhibitory receptor, KIR3DL3, are targets for tumor immunotherapy. HHLA2 has some parallels to the CD80/CD86/CD28/CTLA-4 pathway with a stimulatory receptor, TMIGD2, and an inhibitory receptor, KIR3DL3 [7, 12, 24]. HHLA2 has been shown to be expressed in ccRCC and other tumor types by immunohistochemistry and mRNA expression (9, 10, 12, 16). HHLA2 expression in kidney and lung tumors has been shown to be non-overlapping with PD-L1 expression (12, 14, 25), suggesting that HHLA2 might mediate a mechanism of tumor immune evasion that is independent from PD-1. HHLA2 expression in tumors has been shown in some cases to be associated with worse outcome [11].

A better understanding of the regulation of HHLA2 in tumor cells and within the tumor microenvironment will facilitate translation of these therapeutic agents into the clinic. The loss of HHLA2 expression on tumor cells during in vitro culture has some parallels with PD-L1 expression. Sixty-six percent of squamous cell carcinomas of the head and neck (SCCHN) expressed PD-L1 ex vivo but none of 3 long-term lines expressed PD-L1 [26]. Interferon-γ induced PD-L1 expression on 2 of 3 long-term SCCHN lines [26]; however, we have shown that interferon- γ is not a signal for HHLA2 expression in RCC in vitro [12]. These results emphasize that there are multiple in vivo signals that can enhance different immune inhibitory molecules on tumors. We show that HHLA2 is expressed on primary kidney tumor cells ex vivo by flow cytometry and mRNA expression. HHLA2 expression on tumor cells is progressively lost when cells are cultured over a 4 week period. A498 and 786-O are well established ccRCC tumor cell lines, historically used in many studies of VEGFR directed therapy in immunodeficient mice since there is no syngeneic mouse model of kidney cancer that recapitulates the biology of human ccRCC. Neither A498 or 786-O express HHLA2 in vitro but gain expression when grown as subcutaneous xenografts in NSG immunodeficient mice and lose expression when re-cultured in vitro. This shows that some signal provided by the tumor microenvironment can induce HHLA2 in vivo. While NSG mice do not have an adaptive immune response and the residual innate immune cells in the NSG are considered defective, some murine immune cells are present in these xenografts and F4/80 + myeloid cells can be found in both the A498 and 786-O xenografts. Thus, the signal for HHLA2 induction must be provided by some in vivo signal from the innate immune system, stromal cells, or a microenvironmental condition. Since this signal is from a mouse environment to human cells, it is not species-restricted***.*** A large panel of cytokines and culture conditions, including hypoxia, was screened for the induction of HHLA2 on A498 and 786-O in vitro but no tested condition induced HHLA2. VHL deficiency is a hallmark of clear cell kidney cancer, but not of papillary or chromophobe kidney cancer [27, 28]. VHL mutations were reported in 59.3% of clear cell, 5.2% of papillary, 3.1% of chromophobe carcinomas [29]. However, HHLA2 expression is common on both clear cell and papillary kidney cancer but rare in chromophobe [30]. This says that HHLA2 expression is not at all concordant with VHL loss (clear cell: VHL- HHLA2 + ; papillary: VHL + HHLA2 + ; chromophobe VHL + HHLA2-).

Monocytes and dendritic cells are important innate immune system regulators of the anti-tumor immune response. Analysis of HHLA2 expression in monocytes and dendritic cells shows that among a wide range of cytokines tested, only IL-10 and BMP4, and to a lesser extent, IL-1β and IL-6, modestly enhanced HHLA2 protein and mRNA expression. These results show that HHLA2 expression is differentially regulated in kidney cancer epithelial cells and monocytes. High concentrations of cytokines, particularly IL-10, can induce HHLA2 expression in monocytes but fail to upregulate HHLA2 expression in clear cell kidney cancer cells; however, the tumor microenvironment in NSG mice can induce HHLA2 expression.

As a factor regulating HHLA2 expression, this study examined the regulation of HHLA2 expression in monocytes and DCs, primarily with cytokine-mediated stimuli. We found that IL-10, which plays an important role in the tumor microenvironment, upregulates HHLA2 in these myeloid immune cells. IL-10 has been shown to directly affect the function of antigen-presenting cells by suppressing the expression of MHC and costimulatory molecules [31–33], thereby inducing immunosuppression and tolerance. Conversely, IL-10 was also found to enhance effector function by metabolic reprogramming of terminally exhausted CD8 + T cells and natural killer cells, thereby enhancing anti-tumor immunity [34, 35]. HHLA2 plays a dual role as both an inhibitory and a stimulatory immune checkpoint, and like the IL-10 cytokine, has a dual nature. On the other hand, unlike myeloid immune cells, no induction of HHLA2 by IL-10 was observed in RCC cells, which lacked detectable IL-10R1. Similar to previous studies, in this study we found that HHLA2 was overexpressed in many RCCs, but its expression was attenuated after culture in an in vitro environment. This observation suggests that something about the components present in the tumor microenvironment is important for the maintenance of HHLA2 expression.

## Conclusions

HHLA2 expression in kidney cancer exhibits differential regulation between in vivo and in vitro settings. While HHLA2 is expressed in primary tumor cells and ccRCC xenografts, HHLA2 expression is lost during in vitro culture. Moreover, only IL-10 and BMP4 show modest enhancement of HHLA2 expression in monocytes and dendritic cells, but not in ccRCC tumor cells. These findings highlight the importance of the tumor microenvironment in governing HHLA2 expression. Understanding these regulatory mechanisms is important for developing therapeutic strategies.

## Data Availability

The datasets used and/or analyzed during the current study are available from the corresponding author on reasonable request.

## Abbreviations

HHLA2: Human endogenous retrovirus-H long terminal repeat-associating protein 2
LPS: Lipopolysaccharide
ccRCC: Clear cell renal cell carcinoma
PBMCs: Peripheral blood mononuclear cells
iDCs: Immature DCs
mDCs: Mature DCs
